# Autistic traits modulate frontostriatal connectivity during processing of rewarding faces

**DOI:** 10.1093/scan/nsu010

**Published:** 2014-03-13

**Authors:** Thomas B. Sims, Janina Neufeld, Tom Johnstone, Bhismadev Chakrabarti

**Affiliations:** Centre for Integrative Neuroscience and Neurodynamics, School of Psychology and Clinical Language Sciences, University of Reading, Reading RG6 6AL, UK

**Keywords:** autism, empathy, mimicry, reward, fMRI

## Abstract

Deficits in facial mimicry have been widely reported in autism. Some studies have suggested that these deficits are restricted to spontaneous mimicry and do not extend to volitional mimicry. We bridge these apparently inconsistent observations by testing the impact of reward value on neural indices of mimicry and how autistic traits modulate this impact. Neutral faces were conditioned with high and low reward. Subsequently, functional connectivity between the ventral striatum (VS) and inferior frontal gyrus (IFG) was measured while neurotypical adults (*n* = 30) watched happy expressions made by these conditioned faces. We found greater VS–IFG connectivity in response to high reward *vs* low reward happy faces. This difference was negatively proportional to autistic traits, suggesting that reduced spontaneous mimicry of social stimuli seen in autism, may be related to a failure in the modulation of the mirror system by the reward system rather than a circumscribed deficit in the mirror system.

## INTRODUCTION

Mimicry is an intrinsic part of human interaction. Humans spontaneously and unconsciously mimic the emotional facial expressions of others (Dimberg, 1982). Mimicry occurs in response to facial expressions presented subliminally (Dimberg *et al.*, 2000; Bornemann *et al.*, 2012) and even when participants are explicitly instructed to suppress mimicry (Dimberg *et al.*, 2002). The study of spontaneous facial mimicry is important for social psychology as it can provide a physiological index of affective empathy (Meltzoff and Moore, 2002; Sonnby-Borgstrom *et al.*, 2002).

Complex social processes such as liking (McIntosh, 2006; Likowski *et al.*, 2008; Stel *et al.*, 2010), social competition (Lanzetta and Englis, 1989; Weyers *et al.*, 2009) and group membership (Yabar *et al.*, 2006) are known to modulate spontaneous mimicry. These processes effectively alter the reward value attached to the stimuli suggesting that reward may influence the degree of spontaneous mimicry. A direct test of this proposition is provided by a recent psychophysiological study which found that spontaneous facial mimicry (measured using facial EMG) can be modulated by direct manipulation of the reward value of stimuli (Sims *et al.*, 2012). A link between reward and mimicry is particularly relevant when considering social communication in autism spectrum conditions (ASC). Individuals diagnosed with ASC have been shown to display reduced spontaneous mimicry for the emotional facial expressions of others (McIntosh *et al.*, 2006; Beall *et al.*, 2008; Oberman *et al.*, 2009). There is also evidence that ASC might be further characterized by deficits in social reward sensitivity (Dawson *et al.*, 2002; Kohls *et al.,* 2009; Scott-Van Zeeland *et al.*, 2010), although findings from clinical studies are mixed (Chevallier *et al.*, 2012; Kohls *et al.*, 2012; DeMurie *et al.*, 2011). If autism is associated with atypical modulation of the mirror system (involved in mimicry) by the reward system (involved in ascribing reward values to social stimuli) then this might explain why facial mimicry deficits in ASC are more consistently reported in spontaneous rather than volitional mimicry (McIntosh *et al.*, 2006; Oberman *et al.*, 2009). In support of this possibility, we recently reported that individuals with high autistic traits (measured using the AQ, autism spectrum quotient) do not show differences in spontaneous mimicry of highly rewarding *vs* low rewarding happy faces. This was in contrast to individuals low in AQ, who showed greater spontaneous mimicry for highly rewarding compared with low rewarding happy faces (Sims *et al.*, 2012). This finding suggests that a potential reason why autistic individuals do not engage in spontaneous facial mimicry to the same extent as typically developed individuals could be an atypical modulation of mimicry responses by the reward system. This is consistent with different theoretical models of mimicry (Wang and Hamilton, 2012; Hess and Fischer, 2013).

Previous studies in animals and humans have shown a link between the neural systems involved in processing rewards those involved in mimicry (Kuhn *et al.*, 2010; Lebreton *et al.*, 2012; Losin *et al.*, 2012). A recent single-unit recording study has demonstrated that response of putative ‘mirror’ neurons (i.e. a neuron that typically responds to the observation of another’s goal-directed action, located in the macaque F5 region) is modulated by the reward value associated with the action (Caggiano *et al.*, 2012). The human homologue of the macaque F5 is the pars opercularis region of the inferior frontal gyrus (IFG), which has been strongly implicated in human mimicry (Carr *et al.*, 2003; Lee *et al.*, 2006; Caspers *et al.*, 2010; Likowski *et al.*, 2012). Since the results from the electromyography (EMG) study suggested that the reward value had an impact on the extent of spontaneous mimicry (Sims *et al.*, 2012), we hypothesized that this was instantiated through task-driven modulation of the connectivity between the brain areas involved in processing reward and those involved in mimicry. An evaluative conditioning paradigm, adapted from the EMG study, was used outside the scanner to associate the faces of two actors with different levels of reward value. In a subsequent test phase, participants watched clips of the same actors making happy facial expressions, while functional magnetic resonance imaging (fMRI) data were acquired. We measured the functional connectivity between the IFG (a region involved in mimicry, defined here using a meta-analysis of neuroimaging studies of mimicry) and the ventral striatum (VS, a region involved in reward processing, defined here using a meta-analysis of neuroimaging studies of reward). We predicted that VS–IFG functional connectivity for high reward happy faces would be greater than for low reward happy faces.

Additionally, we assessed individual differences in autistic traits and social interaction using the AQ (Baron-Cohen *et al.*, 2001). Autistic traits are distributed as a continuum across the general population and are known to show identical aetiology across the diagnostic divide (Robinson *et al.*, 2011). This experimental approach of studying autistic traits in neurotypicals thus allows to make inferences about the aetiology of autistic traits without potential confounds from a variety of co-morbid conditions often noted in adults with ASC (e.g. depression, anxiety). The AQ is a 50-item questionnaire measure of autistic traits that consists of two main factors: social interaction and attention to detail (Hoekstra *et al.*, 2008). The subscale of social interaction was of particular interest for this study, because higher scores are thought to be related to lower social reward sensitivity. Based on the findings from our previous study we predicted that the strength of the connectivity between the VS and the IFG would correlate negatively with the participants’ scores on the AQ and in particular with participants’ scores on the subscale of social interaction.

To control for the possibility that differences in VS–IFG connectivity between high *vs* low reward face conditions was not solely driven by higher attention to high reward faces, we also measured task-driven modulation of functional connectivity between the VS and Fusiform Gyrus (FFA), which is strongly associated with the attention to faces and face-like objects (Kanwisher *et al.*, 1997). If participants did attend more to the high reward faces than the low reward faces, we would expect them to show increased VS–FFA functional connectivity in the High Reward Happy *vs* Low Reward Happy conditions. As an additional measure of participant attention we used an eye tracker to record the amount of time that participants spent looking at the emotion expressions made by faces conditioned with high and low rewards.

In our previous facial EMG study we found that reward value of the face had no impact on mimicry for angry faces, i.e. social non-rewards (Sims *et al.*, 2012). Accordingly, we used angry expressions made by high and low reward conditioned faces as a control condition in this experiment. We predicted that there would be no difference in VS–IFG connectivity in response to High Reward Angry *vs* Low Reward Angry faces.

## METHOD

### Participants

In total, 30 participants (17 female) aged between 20 and 36 years (mean = 22.80, s.d. = 4.17) were recruited from the University of Reading campus. Participants received an anatomical image of their brain in exchange for their participation. All participants had normal or corrected-to-normal vision. Ethical approval for the study was obtained from the University Research Ethics Committee of the University of Reading and all participants provided informed consent.

### Stimulus materials

During the conditioning phase, stimuli consisted of static images of two target faces (one male and one female) with neutral facial expressions. In the test phase, stimuli used consisted of four 4000 ms video clips showing dynamic emotional facial expressions made by the same two target identities. Dynamic expressions were used instead of still pictures as they have been shown to be more ecologically valid (Hess and Blairy, 2001). All stimuli were selected from the ‘Mindreading set’ (Baron-Cohen *et al.*, 2004, available at www.jkp.com/mindreading). These stimuli have been shown to have high inter-rater reliability and external validity (Golan and Baron-Cohen, 2006; Golan *et al.*, 2006). All stimuli were displayed using E-Prime 2.0 (Psychology Software Tools, PA, USA).

### Procedure

The procedure closely resembled that which was described previously in Sims *et al.* (2012) with adjustments made to make it more suitable for fMRI scanning. Prior to scanning, participants were seated at a distance of 55 cm from a Viewsonic VE510s monitor (colour TFT active matrix XGA LCD 30.5 × 23 cm) and introduced to the implicit evaluative conditioning task. The instructions for all tasks were presented on the monitor and also read aloud by the experimenter. After a short practice session consisting of eight trials, the experimenter left the room while the participants performed a conditioning task and returned afterwards to introduce the test phase. The participants had a practise session consisting of six trials, before being positioned inside the MRI scanner. The test phase stimuli were presented using NordicNeuroLab’s VisualSystem (Nordic Neurolab Inc, WI, USA), with a OLED display of 30° horizontal and 23° vertical (800 × 600 pixels). After completion of the test phase participants were debriefed and dismissed.

### Conditioning phase

**Fig. 1:**
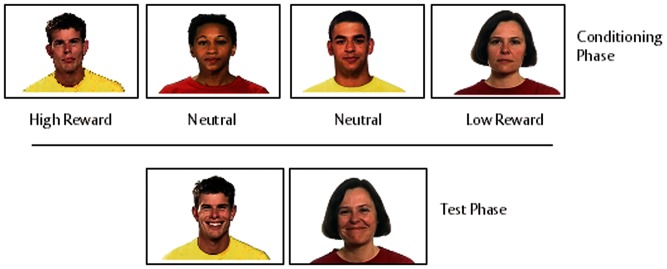
Stimulus material. The top panel shows an example of the four actors that were implicitly conditioned (30 repetitions) with four probabilities in the conditioning phase (90% win, 60% win, 60% loss and 90% loss). At the start of each trial participants were presented with two standard playing cards. The first card was face up and the second card was face down. Participants used one of the two keys on the keyboard to predict whether they believed the second card would be of greater or lesser value than the first card. There was no time limit in which the participants were required to respond. If they were correct in their prediction, then they had won 25p. If they were incorrect, they lost 20p. This feedback was displayed for 4000 ms. If the cards were of equal value then the participant neither won nor lost money. The outcome of all of the trials, regardless of participant response, was pre-determined and the feedback adjusted accordingly. This was followed by the test phase (bottom panel) where participants observed dynamic happy and angry expressions made by the four actors (eight presentations of each clip). Each clip lasted 4 s and was preceded by a 1 s fixation cross. A blank screen was presented for 1 s between each trial.

The conditioning phase took place outside of the MRI scanner. In each trial a target face with a neutral expression (Figure 1) appeared alongside a card guessing game, as described in Sims *et al.* (2012). At the start of each trial participants were presented with two standard playing cards. The first card was face up, and the second card was face down. Participants used one of two keys on the keyboard to predict whether the second card would be of greater or lesser value than the first card. There was no time limit for the response. For each correct prediction participants won 25p; for each incorrect guess they lost 20p. If the cards were of equal value then the participant neither won nor lost money (‘tie’ trials). A feedback about the amount of money won or lost in each trial was displayed for 4000 ms after the participant’s response. The outcome of all of the trials, regardless of participant response, was pre-determined and the feedback adjusted accordingly. The reward level attributed to each target face was manipulated by adjusting the number of trials that were won or lost in the presence of each face. In the High Reward condition, participants won 90% of the trials that were paired with the associated face; in the Low Reward condition participants lost 90% of trials associated with the face. The faces in the high and low reward conditions, respectively, were counterbalanced across participants. In order to disguise the underlying structure of the game, two further faces (1 male, 1 female) were paired with half of the trials. Participants won and lost an equal number of trials associated with these two faces. These two faces did not appear in the test phase of the study. Each of the four faces (two target faces and two additional faces) was presented a total of 30 times. In total, the conditioning phase consisted of 120 trials.

The presence of the faces alongside the cards was explained by informing the participants that the second half of the study would involve a simple memory task.

### Test phase

During the test phase participants were presented with 4000 ms video clips of the conditioned faces making emotional facial expressions. There were two expressions for each face, happy or angry. Each clip was preceded by a fixation cross, the duration of which was jittered (mean* = *1324.57 ms, s.d. = 330.66). The duration of jitter and the order of presentation of stimuli were designed to maximize power for estimating the contrast of interest using OptSeq (http://www.surfer.nmr.mgh.harvard.edu/optseq).

Randomly distributed throughout the presentation of the target clips there were 15 clips which contained an emotion expression (happy/angry) made by an ‘oddball’ face (i.e. an actor that was not present in the conditioning phase). Ostensibly the participants were engaged in a memory task to spot the novel faces. They were asked to press a button on a button box that they held in their right hand each time an oddball face was presented. Participants did not receive any feedback for correct or incorrect responses. The task served solely as a means of ensuring that the participants were paying attention to the target faces. The test phase consisted of a total of 175 video clips; 160 target clips (40 for each of the four conditions) and 15 oddball clips. The test phase was split into two runs of equal length to avoid fatigue and diminishment of concentration. The first run consisted of 88 (80 target clips, 8 oddball clips) and run 2 consisted of 87 clips (80 target clips, 7 oddball clips). Data from only the first run only is presented in this article, because (i) the number of test phase trials per condition are comparable with our earlier EMG study and (ii) the second run was associated with extinction of the learnt rewards (due to 20 more presentations of the conditioned stimuli without the reinforcing unconditioned stimuli).

### Regions of interest

**Fig. 2:**
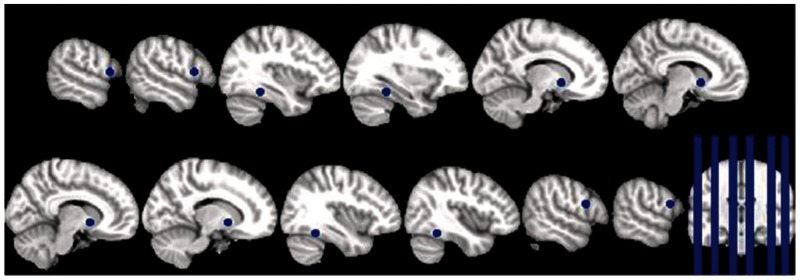
Pre-defined regions of interest. ROIs within IFG, VS and FFA were identified using coordinates of published meta-analyses of relevant neuroimaging studies. The pickatlas tool in SPM was used to draw spheres with 5 mm radius around the centre coordinates of the selected ROIs. The ROIs were defined in the right and left IFG reported in a meta-analysis of mimicry studies by Caspers *et al.* (2010) [right (58, 10, 20); left (−56, 12, 9)], right and left VS reported in a meta-analysis of neuroimaging studies of reward by Liu *et al.* (2011) [right (12, 8, −4); left (−10, 10, −4)] and right and left FFA reported the meta-analysis of emotional face processing in Fusar-Poli *et al.* (2009) [right (40, −49, 18); left (−35, −42, 17)].

Regions of interest (ROIs) within IFG, VS and FFA were identified using coordinates of published meta-analyses of relevant neuroimaging studies. The pickatlas tool in SPM was used to draw spheres with 5 mm radius around the centre coordinates of the selected ROIs. The ROIs were defined in the right and left IFG reported in a meta-analysis of mimicry studies by Caspers *et al.* (2010) [right (58, 10, 20); left (−56, 12, 9)], right and left VS reported in a meta-analysis of neuroimaging studies of reward by Liu *et al.* (2011) [right (12, 8, −4); left (−10, 10, −4)]; and right and left FFA reported the meta-analysis of emotional face processing in Fusar-Poli *et al.* (2009) [right (40, −49, −18); left (−35, −42, −17)] (Figure 2).

### Trait measurements

Prior to their participation, 25 of the 30 participants completed the AQ. Scores on the AQ ranged between 9 and 29 (mean = 16.84, s.d. = 5.35). No participant scored >32 on the full AQ, which has been found to be a reliable threshold score for a potential clinical diagnosis of ASC.

### FMRI analysis

#### Scanning and pre-processing

Participants were scanned in a 3T Siemens TIM Trio MRI scanner with 12 channel head coil {28 inter-leaved, 2.5 mm thick axial slices [repetition time (TR) = 1500 ms; echo time (TE) 28ms]}. DICOM files were converted to NIfTI data image files using dcm2nii in MRICron. FMRI data processing was carried out using FEAT (FMRI Expert Analysis Tool) version 5.98, part of FSL (FMRIB’s Software Library, www.fmrib.ox.ac.uk/fsl). The following pre-statistics processing was applied; motion correction using MCFLIRT (Jenkinson *et al.*, 2002); interleaved slice-timing correction using Fourier-space time-series phase-shifting; non-brain removal using BET (Smith, 2002); spatial smoothing using a Gaussian kernel of FWHM 5 mm; grand-mean intensity normalization of the entire 4D dataset by a single multiplicative factor; highpass temporal filtering (Gaussian-weighted least-squares straight line fitting, with σ = 50.0 s). Time-series statistical analysis was carried out using FILM with local autocorrelation correction (Woolrich *et al.*, 2001). Registration to high resolution structural and standard space images was carried out using FLIRT (Jenkinson and Smith, 2001).

#### Psychophysiological interaction analysis

Time-courses for both of the seed regions (i.e. left VS and right VS) for the entire run were extracted independently using FSL. Interactions were used as regressors in the psychophysiological interaction (PPI) analysis at the first level; four PPIs were computed in total (High Reward Right VS; Low Reward Right VS; High Reward Left VS; Low Reward Left VS). Main task regressors were also included at the first level. This was followed by extracting the mean *z*-stat for each PPI term relative to baseline, for the left and right IFG ROIs as defined earlier for each hemisphere. Paired sample *t*-tests were conducted to compare VS–IFG functional connectivity in the High Reward Happy *vs* Low Reward Happy conditions. An identical analysis was done to estimate the task-related changes in connectivity of VS and FFA separately for each hemisphere.

Both VS–IFG and VS–FFA functional connectivity for [High Reward Happy–Low Reward Happy] condition observed by PPI analysis was correlated with participants’ AQ scores. One data point was removed from the correlation analysis as the AQ score was more than 2 s.d. from the group mean {and a leverage value of 0.21, greater than the cutoff value of 0.17 [derived using the formula (2*k* + 2)/*n*, where *k* is the number of predictor variables and *n* is the sample size]}.

#### Functional connectivity analysis using β-series correlation

In order to verify the results of the PPI analysis, an additional functional connectivity analysis was conducted, using a β-series correlation approach (Rissman *et al.*, 2004). In this method parameter estimates (*β*-values) are calculated for each single trial. For each task condition the mean *β*-values of a seed region are then correlated across trials with the *β*-values of each voxel of the brain, resulting in condition-specific seed correlation maps. In contrast to PPI, the approach is model-free and the direction of influence of one neural system on another is not specified in this analysis.

Data pre-processing was conducted using SPM8 (http://www.fil.ion.ucl.ac.uk/spm) using parameters identical to the FSL-based analysis. After slice-timing correction, images were realigned to the first volume to corrected for inter-scan movements with a least squares approach and a rigid body spatial transformation to remove artefacts. The mean image obtained from the realignment process was co-registered to a T2 anatomical scan of each participant. Realigned images were normalized to the EPI-derived MNI template (ICBM 152, Montreal Neurological Institute), using the co-registered mean image as source image, resulting in a voxel size of 2 × 2 × 2 mm. Normalized images were finally smoothed with a Gaussian kernel of 5 mm full-width half-maximum and filtered with a high-pass filter of 128 s.

To investigate the functional connectivity between the VS and the IFG during visual processing of faces associated with high and low learned reward value, β-series correlation was performed. Haemodynamic responses were modelled for each trial as separate covariate of interest for each individual subject, using a general linear model (GLM). Estimated movement parameters were included in the model to minimize signal-correlated motion effects. Parameter estimates (*β*-values) were extracted to form a set of condition-specific β-series for each participant and each presented stimulus. The seed regions were defined as sphere with 5 mm radius around the centre of mass of clusters in left and right VS as defined earlier. β-Series of each seed were averaged across voxels within the critical region and correlated with β-series of every other voxel in the whole brain. For each participant, maps of correlation coefficients were calculated for each condition (first level analysis) and normalized by using an arc-hyperbolic tangent transform for further statistical inference.

In the second step of the analysis, paired *t*-tests were conducted for each of the two seeds (left and right VS) to examine connectivity differences between processing of faces with high *vs* low reward value. This was followed by a hypothesis-driven ROI analysis by extracting the contrast value from the two ROIs located in the left and right IFG as described before. ROI analysis was conducted using MarsBar 0.42 (Brett *et al.*, 2002).

### Eye gaze tracking

Participant’s eye movements were recorded using a ViewPoint EyeTracker^®^ (Arrington Res. Inc., AZ, USA). Mean visit duration was calculated for each condition. Visit duration represented the total time during a single trial that eye-gaze was detected anywhere within the area of the screen occupied of the stimuli faces. Unfortunately, data from 16 (of the original 30) participants had to be excluded from the eye-gaze analysis due to technical problems, resulting in eye-gaze tracking data being retained for 14 participants.

## RESULTS

All statistical tests in the results section are one-tailed in keeping with the directional nature of the hypotheses.

### Behavioural data

In the test phase the oddball task was performed at ceiling with 100% accuracy, with none of the 30 participants making any mistakes during the task. This indicated that the participants were attending to the stimuli.

### VS–IFG functional connectivity

**Fig. 3:**
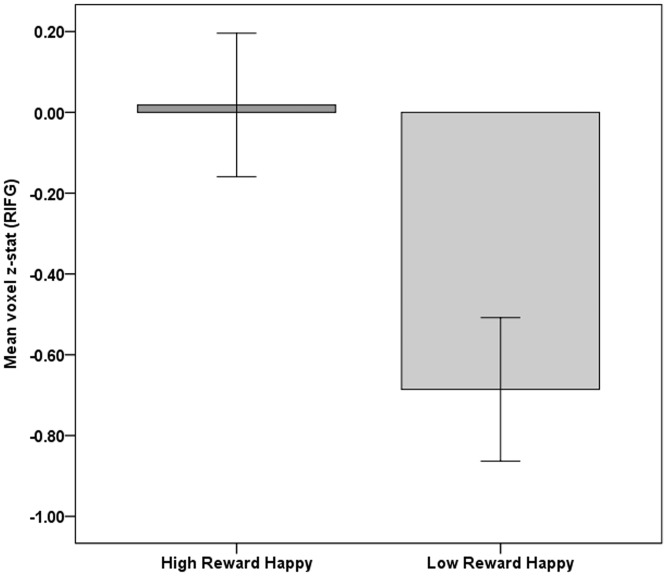
Functional VS–IFG connectivity (PPI) during the High and Low Reward Happy conditions. Participants were conditioned to different levels of reward to two different neutral faces. Participants then viewed 4000 ms movie clips of the same two faces making happy facial expressions. A psychophysiological interaction analysis was performed with a physiological seed located in the right ventral striatum. The y-axis represents the mean *z*-stat relative to baseline for voxels inside the right IFG ROI. The error bars depict ±1 within-subjects standard error of mean (calculated using the method as described in Loftus and Masson (1994).

In line with the results from the facial EMG study, we predicted that the connectivity between VS and IFG would be stronger in the High Reward Happy *vs* Low Reward Happy condition. Paired sample *t*-tests revealed that the VS–IFG connectivity (PPI) in the right hemisphere was significantly greater in High Reward Happy *vs* Low Reward Happy condition [*t*(29) = 1.913, *P* = 0.033, *d* = 0.357). There was no significant difference in VS–IFG connectivity in the left hemisphere between the two conditions; [*t*(29) = 0.238, *P* = 0.406, *d* = 0.044]. This finding was further confirmed by an independent β-series correlation analysis [right hemisphere: *t*(29) = 1.81, *P* = 0.038; left hemisphere: *t*(29) = −0.17, *P* = 0.566] (Figure 3).

As predicted, there was no significant difference in co-activation between VS and IFG in the High Reward Angry *vs* Low Reward Angry condition [PPI right hemisphere: *t*(29) = 0.467, *P* = 0.322, *d* = 0.085; left hemisphere: *t*(29) = −1.044, *P* = 0.152, *d* = −0.191, β-series correlation: right hemisphere: *t*(29) = 0.700, *P* = 0.243; left hemisphere: *t*(29) = 0.360, *P* = 0.361].

### VS–FFA functional connectivity

Paired sample *t*-tests confirmed that there was no significant difference in VS–FFA functional connectivity between the High Reward Happy *vs* Low Reward Happy conditions in either the right or left hemispheres for both PPI analysis; right *t*(29) = 0.310, *P* = 0.380, *d* = 0.057; left *t*(29) = −1.329, *P* = 0.097, *d* = −0.244 as well as β-series correlation analysis [right: *t*(29) = −1.41, *P* = 0.081; left: *t*(29) = 0.58, *P* = 0.281].

### Eye-gaze analysis

Paired sample *t*-tests confirmed that there was no significant difference in the mean visit duration for faces in the High Reward Happy *vs* Low Reward Happy conditions [*t*(13) = −0.580, *P* = 0.571, *d* = −0.157].

### Correlation analysis

**Fig. 4:**
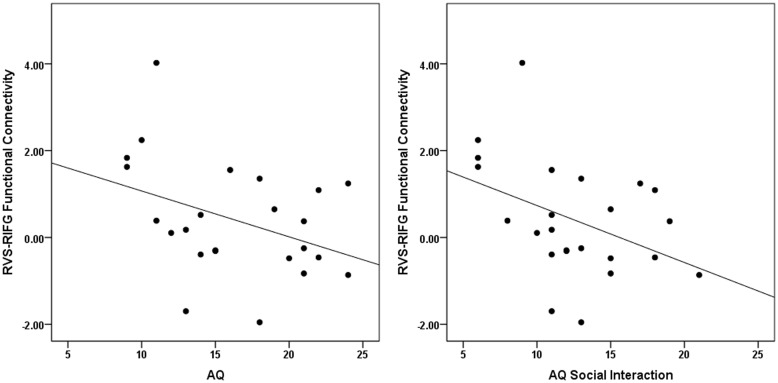
Correlations between right VS–IFG functional connectivity (PPI) and participants scores AQ and AQ social interaction subtrait. Participants completed the Autism Spectrum Quotient (AQ) which measures autistic traits in the general population. The x-axis represents participant scores on the AQ (left panel) and the AQ subtrait of social interaction (right panel). The y-axis in all plots indicates the RVS–RIFG functional connectivity [High Reward–Low Reward].

We predicted that the VS–IFG functional connectivity for (High Reward–Low Reward) Happy condition would be inversely proportional to autistic traits. Correlation analysis confirmed that the VS–IFG functional connectivity in the right hemisphere for (High Reward–Low Reward) Happy condition correlated negatively with participants’ scores on the AQ *r*(24) = −0.384, *P* = 0.032 and negatively with the subtraits of social interaction *r*(24) = −0.409, *P* = 0.024 (Figure 4).

There was no significant correlation between VS–FFA functional connectivity in the right hemisphere for (High Reward–Low Reward) Happy faces and either AQ scores *r*(24) = −0.104, *P* = 0.315 or social interaction scores *r*(24) = −0.100, *P* = 0.321.

## DISCUSSION

In this fMRI study, we followed up our previous facial EMG study, which found that more rewarding happy faces were associated with greater spontaneous mimicry (Sims *et al.*, 2012). In this study in a new sample of volunteers, we tested if the functional connectivity between brain regions involved in processing rewards and those involved in mimicry changed significantly when participants watched happy expressions of faces associated with High *vs* Low Reward. Specifically, functional connectivity between the VS and IFG was measured while the participants watched happy expressions of faces conditioned with High *vs* Low Reward. Two separate analyses of functional connectivity conducted using PPI and β-series correlation analyses were found to support our prediction. Specifically, co-variation in the right VS and right IFG was significantly stronger in the High Reward Happy *vs*. Low Reward Happy condition. As the VS and the IFG are known to play central roles in reward processing and mimicry, respectively, these findings provide direct evidence of a functional link between these systems in the human brain. Importantly, we observed that the extent of this co-variation in the high reward condition was modulated by individual difference in autistic traits in general and in particular, the AQ sub-component of social interaction. These findings add further evidence for a link between mimicry and reward system as indicated previously by EMG data (Sims *et al.*, 2012). Notably, the effect size of the correlation of the AQ with the difference in the extent of spontaneous mimicry of [High Reward–Low Reward] Happy faces (*r* = –0.375) was very similar to the effect size of the correlation of AQ with VS–IFG connectivity in response to (High Reward–Low Reward) Happy faces (*r* = −0.384). This provides convergent validity of these results across two different experimental technique and independent samples. One aspect of the current findings that requires further investigation, is whether the greater VS–IFG functional connectivity for (High Reward–Low Reward) Happy faces in participants with lower AQ scores is driven largely by increased VS–IFG coupling in the high reward condition in this group or whether it is driven by reduced VS–IFG coupling in the low reward condition.

It is unlikely that the above reported difference in VS–IFG connectivity was the result of differences in the attention to the faces between the two reward conditions as there was no evidence of increased VS–FFA functional connectivity for high reward happy faces in the current study. Indeed there was a very slight trend towards greater VS–FFA functional connectivity for low reward happy faces. This finding receives additional support from the eye tracking data which showed that there was no difference in the average time that participants spent looking at happy faces in the two reward conditions. However, eye tracking data was only available for a small subset of the participants (*n* = 14) and so the null findings could result from low statistical power in this analysis. There was no correlation between VS–FFA functional connectivity for (High Reward–Low Reward) Happy faces and either scores on the AQ or the subscore of social interaction. Therefore, it would seem unlikely that evidence of greater VS–IFG functional connectivity in lower AQ participants be attributed to individual differences in attention.

There was no evidence of a modulation of VS–IFG connectivity by the reward condition for angry faces. This is in line with our previous study where we could show that mimicry for angry faces is unaffected by reward value. We speculate that reinforcement of spontaneous mimicry during social interaction—and therefore the reinforcement of social interactions in general—depends on functional connectivity between the brain’s reward and mimicry systems. Conditions such as ASC, which may be marked by both an impaired response to social rewards (Dawson *et al.*, 2005; Kohls *et al.*, 2009; Scott-Vanzeeland *et al.*, 2010; —but also see DeMurie *et al.*, 2011) as well as reduced spontaneous mimicry of social stimuli (McIntosh *et al.*, 2006; Beall *et al.*, 2008), could potentially constitute a disruption to this reward/mimicry link. A potential disruption of this link might be able to explain why mirror systems are not brought ‘online’ spontaneously during social interaction. This speculation is supported by our finding that the strength of the reward/mimicry connectivity was inversely proportional to autistic traits; which replicates our facial EMG results both in direction and magnitude.

It should be noted that studies which report impaired spontaneous mimicry in ASC largely tend to use facial imitation; findings from studies using hand imitation are less consistent (see Spengler *et al.*, 2010). It has been proposed that the development of face and hand imitation might rely on different processes. Although infants are able to visually match their own hand movements to those of others, they have no visual reference for their own facial expressions. It is therefore suggested that facial imitation must rely on processes that are, in part at least, genetically pre-wired (Casile *et al.*, 2011). The consistent finding of a deficit in spontaneous mimicry of faces, but not of hands, in ASC participants suggests that deficits in the ASC mimicry mechanisms maybe limited to the pre-wired system.

Just over half of the participants in this study were female (*n* = 17). Although there has been increased interest in the study of autism in females (e.g. Lai *et al.*, 2011), most research still indicates that the condition is more prevalent in males (Fombonne, 2005). A gender comparison is beyond the scope of this study. However, given that males are known to score significantly higher than females on the AQ, we predict that the reward–mimicry link would be weaker in male participants compared with females. This prediction makes the assumption that the relationship between the reward–mimicry link and AQ is the same across genders and this too needs to be systematically investigated in a future study.

As spontaneous facial mimicry is regarded as a marker of empathy, we argue that the current set of findings provides further evidence for a link between the brain reward and empathy systems. This finding is in line with those from animal research which have demonstrated the crucial role that reward plays in social behaviours such as pair bonding and maternal bonding (Keverne and Curley, 2004), whereas evidence from pharmacological and gene studies have shown that blocking reward system results in a range of impairments of social behaviour (McGregor *et al.*, 1996; Moles *et al.*, 2004).

Although this proposed link has not been fully explored in humans, certain pathological conditions associated with deficits in the dopaminergic system, such as Parkinson’s disease, have also known to have reduced social functioning (Lawrence *et al.*, 2007). Reward-related brain regions have been shown to play a role in empathic processing in children (Brink *et al.*, 2011), while altered functioning of reward regions in the brain have been recorded in individuals diagnosed with anti-social personality disorder (Vollm *et al.*, 2010). The notion of a reward–empathy link in humans has received support from a recent study separate coordinate-based meta-analyses performed on reward and empathy functional imagery studies, which found overlapping activation in a number of brain regions, including the VS and IFG (O’Connell *et al.*, 2013).

In conclusion, we found that co-activation of right VS and the right IFG was greater when participants viewed happy expressions made by faces previously conditioned with high reward *vs* low reward. In view of the critical role of the IFG in mimicry, we argue that this finding provides evidence of a functional link between the brain’s reward and mimicry systems. As mimicry is a component of empathy, we speculate that a disruption of this link could potentially point to the aetiology of some of the social behavioural deficits seen in conditions such as ASC. This speculation is supported by our finding that the difference in connectivity between VS and IFG in response to highly rewarding *vs* low rewarding happy faces was weaker in participants who scored higher in autistic traits. This set of results suggest that atypical connectivity between brain regions involved in reward and mimicry in individuals with high autistic traits (e.g. ASC) could explain why these individuals do not show spontaneous mimicry of social stimuli to the same extent as individuals within the typically developed population. This suggestion provides a theoretical bridge between studies that suggest a mirror system deficit in autism (Dapretto *et al.*, 2005; McIntosh *et al.*, 2006; Beall *et al.*, 2008) and those that do not (Bird *et al.*, 2007; Dinstein *et al.*, 2010).

## Conflict of Interest

None declared.

## References

[nsu010-B1] Baron-Cohen S, Golan O, Wheelwright S, Hill J (2004). Mindreading: The Interactive Guide to Emotions.

[nsu010-B2] Baron-Cohen S, Wheelwright S, Skinner R, Martin J, Clubley E (2001). The Autism-Spectrum Quotient (AQ): Evidence from Asperger Syndrome/high-functioning autism, males and females, scientists and mathematicians. Journal of Autism and Developmental Disorders.

[nsu010-B3] Beall PM, Moody EJ, McIntosh DN, Hepburn SL, Reed CL (2008). Rapid facial reactions to emotional facial expressions in typically developing children and children with autism spectrum disorder. Journal of Experimental Child Psychology.

[nsu010-B4] Bird G, Leighton J, Press C, Heyes C (2007). Intact automatic imitation of human and robot actions in autism spectrum disorders. Proceedings of the Royal Society.

[nsu010-B5] Bornemann B, Winkielman P, van der Meer E (2012). Can you feel what you do not see? Using internal feedback to detect briefly presented emotional stimuli. International Journal of Psychophysiology.

[nsu010-B6] Brett M, Anton J, Valabregue R, Poline J (2002). Region of interest analysis using an SPM toolbox.

[nsu010-B7] Brink TT, Urton K, Held D (2011). The role of orbitofrontal cortex in processing empathy stories in 4- to 8-year-old children. Frontiers in Psychology.

[nsu010-B8] Caggiano V, Fogassi L, Rizzolatti G, Casile A, Giese M, Thier P (2012). Mirror neurons encode the subjective value of an observed action. Proceedings of the National Academy of Sciences of the USA.

[nsu010-B9] Carr L, Iacoboni M, Dubeau M-C, Mazziotta JC, Lenzi GL (2003). Neural mechanisms of empathy in humans: a relay from neural systems for imitation to limbic areas. Proceedings of the National Academy of Sciences of the USA.

[nsu010-B10] Casile A, Caggiano V, Ferrari PF (2011). The mirror neuron system: a fresh view. The Neuroscientists: a review journal bringing neurobiology, neurology and psychiatry.

[nsu010-B11] Caspers S, Zilles K, Laird AR, Eickhoff SB (2010). ALE meta-analysis of action observation and imitation in the human brain. NeuroImage.

[nsu010-B12] Chevallier C, Kohls G, Troiani V, Brodkin ES, Schultz RT (2012). The social motivation theory of autism. Trends in cognitive sciences.

[nsu010-B13] Dapretto M, Davies MS, Pfeifer JH (2005). Understanding emotions in others: mirror neuron dysfunction in children with autism spectrum disorders. Nature Neuroscience.

[nsu010-B14] Dawson G, Munson J, Estes A (2002). Neurocognitive function and joint attention ability in young children with autism spectrum disorder versus developmental delay. Child Development.

[nsu010-B15] Dawson G, Webb SJ, McPartland J (2005). Understanding the nature of face processing impairment in autism: insights from behavioural and electrophysiological studies. Developmental Neuropsychology.

[nsu010-B16] Demurie E, Roeyers H, Baeyens D, Sonuga-Barke E (2011). Common alterations in sensitivity to type but not amount of reward in ADHD and autism spectrum disorders. Journal of Child Psychology and Psychiatry, and Allied Disciplines.

[nsu010-B17] Dimberg U (1982). Facial reactions to facial expressions. Psychophysiology.

[nsu010-B18] Dimberg U, Thunberg M, Elmehed K (2000). Unconscious facial reactions to emotional facial expressions. Psychological Science.

[nsu010-B19] Dimberg U, Thunberg M, Grunedal S (2002). Facial reactions to emotional stimuli: Automatically controlled emotional responses. Cognition and Emotion.

[nsu010-B20] Dinstein I, Thomas C, Humphreys K, Minshew N, Behrmann M, Heeger DJ (2010). Normal movement-selectivity in autism.

[nsu010-B21] Fombonne E, Volkmar F, Paul R, Klin A, Cohen D (2005). Epidemiological studies of pervasive developmental disorder. Handbook of Autism and Pervasive Developmental Disorders.

[nsu010-B22] Fusar-Poli P, Placentino A, Carletti F (2009). Laterality effect on emotional faces processing: ALE meta-analysis of evidence. Neuroscience Letters.

[nsu010-B23] Golan O, Baron-Cohen S (2006). Systemizing empathy: teaching adults with Asperger’s syndrome/high functioning autism to recognize emotions using interactive multimedia. Development and Psychopathology.

[nsu010-B24] Golan O, Baron-Cohen S, Hill J (2006). The Cambridge Mindreading (CAM) Face-Voice Battery: Testing complex emotion recognition in adults with and without Asperger’s syndrome. Journal of Autism and Developmental Disorders.

[nsu010-B25] Hess U, Blairy S (2001). Facial mimicry and emotional contagion to dynamic emotional facial expressions and their influence on decoding accuracy. International Journal of Psychophysiology.

[nsu010-B26] Hess U, Fischer A (2013). Emotional mimicry as social regulation. Personality and Social Psychology Review.

[nsu010-B27] Hoekstra R, Bartels M, Cath DC, Boomsma DI (2008). Factor structure, reliability and criterion validity of the Autism-Spectrum Quotient (AQ): a study in Dutch population and patient groups. Journal of Autism and Developmental Disorders.

[nsu010-B28] Jenkinson M, Bannister P, Brady M, Smith S (2002). Improved optimization for the robust and accurate linear registration and motion correction of brain images. NeuroImage.

[nsu010-B29] Jenkinson M, Smith S (2001). A global optimisation method for robust affine registration of brain images. Medical Image Analysis.

[nsu010-B30] Kanwisher N, McDermott J, Chun MM (1997). The fusiform face area: a module in human extrastriate cortex specialized for face perception. The Journal of Neuroscience.

[nsu010-B31] Keverne EB, Curley JP (2004). Vasopressin, oxytocin and social behaviour. Current Opinion in Neurobiology.

[nsu010-B32] Kohls G, Chevallier C, Troiani V, Schultz RT (2012). Social “wanting” dysfunction in autism: neurobiological underpinnings and treatment implications. Journal of Neurodevelopmental Disorders.

[nsu010-B33] Kohls G, Peltzer J, Herpertz-Dahlmann B, Konrad K (2009). Differential effects of social and non-social reward on response inhibition in children and adolescents. Developmental Science.

[nsu010-B34] Kühn S, Müller BC, van Baaren RB, Wietzker A, Dijksterhuis A, Brass M (2010). Why do I like you when you behave `like me? Neural mechanisms mediating positive consequences of observing someone being imitated. Social Neuroscience.

[nsu010-B35] Lai MC, Lombardo MV, Pasco G (2011). A behavioral comparison of male and female adults with high functioning autism spectrum conditions. PLoS One.

[nsu010-B36] Lawrence AD, Goerendt IK, Brooks DJ (2007). Impaired recognition of facial expressions of anger in Parkinson’s disease patients acutely withdrawn from dopamine replacement therapy. Neuropsychologia.

[nsu010-B37] Lanzetta JT, Englis BG (1989). Expectations of cooperation and competition and their effects on observers’ vicarious emotional responses. Journal of Personality and Social Psychology.

[nsu010-B38] Lebreton M, Kawa S, Forgeot d’Arc B, Daunizeau J, Pessiglione M (2012). Your goal is mine: unraveling mimetic desires in the human brain. The Journal of Neuroscience.

[nsu010-B39] Lee TW, Josephs O, Dolan RJ, Critchley HD (2006). Imitating expressions: emotion-specific neural substrates in facial mimicry. Social Cognitive and Affective Neuroscience.

[nsu010-B40] Likowski KU, Mühlberger A, Gerdes ABM, Wieser MJ, Pauli P, Weyers P (2012). Facial mimicry and the mirror neuron system: simultaneous acquisition of facial electromyography and functional magnetic resonance imaging. Frontiers in Human Neuroscience.

[nsu010-B41] Likowski KU, Mühlberger A, Seibt B, Pauli P, Weyers P (2008). Modulation of facial mimicry by attitudes. Journal of Experimental Social Psychology.

[nsu010-B42] Liu X, Hairston J, Schrier M, Fan J (2011). Common and distinct networks underlying reward valence and processing stages: a meta-analysis of functional neuroimaging studies. Neuroscience and Biobehavioral Reviews.

[nsu010-B43] Loftus GR, Masson MEJ (1994). Using confidence intervals in within-subject designs. Psychonomic Bulletin & Review.

[nsu010-B44] Losin EAR, Iacoboni M, Martin A, Dapretto M (2012). Own-gender imitation activates the brain’s reward circuitry. Social Cognition and Affective Neuroscience.

[nsu010-B45] McIntosh DN (2006). Spontaneous facial mimicry, liking and emotional contagion. Polish Psychological Bulletin.

[nsu010-B46] McIntosh DN, Reichmann-Decker A, Winkielman P, Wilbarger JL (2006). When the social mirror breaks: deficits in automatic, but not voluntary, mimicry of emotional facial expressions in autism. Developmental Science.

[nsu010-B47] McGregor IS, Dastur FN, McLellan RA, Brown RE (1996). Cannabinoid modulation of rat pup ultrasonic vocalizations. European Journal of Pharmacology.

[nsu010-B48] Meltzoff AN, Moore MK (2002). Imitation, memory, and the representation of persons. Infant Behavior and Development.

[nsu010-B49] Moles A, Kieffer B, D’Amato F (2004). Deficit in attachment behavior in mice lacking the µ-opioid receptor gene. Science.

[nsu010-B50] Oberman LM, Winkielman P, Ramachandran VS (2009). Slow echo: facial EMG evidence for the delay of spontaneous, but not voluntary, emotional mimicry in children with autism spectrum disorders. Developmental Science.

[nsu010-B51] O’Connell G, Sims TB, Chakrabarti B (2013). Meta-analysis of neural correlates of empathy and reward: Evidence for an overlap.

[nsu010-B52] Rissman J, Gazzaley A, D’Esposito M (2004). Measuring functional connectivity during distinct stages of a cognitive task. NeuroImage.

[nsu010-B53] Robinson EB, Koenen KC, McCormick MC (2011). Evidence that autistic traits show the same etiology in the general population and at the quantitative extremes (5%, 2.5%, and 1%). Archives of General Psychiatry.

[nsu010-B54] Scott-Van Zeeland AA, Dapretto M, Ghahremani DG, Paldrack RA, Bookheimer SY (2010). Reward processing in autism. Autism Research.

[nsu010-B55] Sims TB, Van Reekum CM, Johnstone T, Chakrabarti B (2012). How reward modulates mimicry: EMG evidence of greater facial mimicry of more rewarding happy faces. Psychophysiology.

[nsu010-B56] Smith SM (2002). Fast robust automated brain extraction. Human Brain Mapping.

[nsu010-B57] Sonnby-Borgström M, Jonsson P, Svensson O (2002). Emotional empathy as related to mimicry reactions at different levels of information processing. Journal of Nonverbal Behaviour.

[nsu010-B58] Spengler S, Bird G, Brass M (2010). Hyperimitation of actions is related to reduced understanding of others’ minds in autism spectrum conditions. Biological Psychiatry.

[nsu010-B59] Stel M, van Baaren RB, Blascovich J (2010). Effects of *a priori* liking on the elicitation of mimicry. Experimental Psychology.

[nsu010-B60] Völlm B, Richardson P, McKie S (2010). Neuronal correlates and serotonergic modulation of behavioural inhibition and reward in healthy and antisocial individuals. Journal of Psychiatric Research.

[nsu010-B61] Wang Y, Hamilton AFDC (2012). Social top-down response modulation (STORM): a model of the control of mimicry in social interaction. Human Neuroscience.

[nsu010-B62] Weyers P, Muhlberger A, Kund A, Hess U, Pauli P (2009). Modulation of facial reactions to avatar emotional faces by nonconscious competition priming. Psychophysiology.

[nsu010-B63] Woolrich M, Ripley B, Brady J, Smith S (2001). Temporal autocorrelation in univariate linear modelling of FMRI data. NeuroImage.

[nsu010-B64] Yabar Y, Johnston L, Miles L, Peace V (2006). Implicit behavioral mimicry: investigating the impact of group membership. Journal of Nonverbal Behaviour.

